# Institutional Needs

**Published:** 1914-04-25

**Authors:** 


### Institutional Needs.

### "SECWAY."

A food comprising "pure sweet whey powder," whether regarded as a food complete in itself or merely as a milk modifier, is doubly sure of attention in these days when the milk question is seen to be rather further from than nearer to solution than formerly. "Secway" is the trade name of such a product, manufactured by Casein, Limited, Battersea, who claim for it a larger percentage of whey albumens than is found in any other product on the market. The reason why whey cannot ordinarily be employed as a complete food is, of course, the amount of water accompanying it, but the makers have endeavoured to overcome this by providing in "Secway" a powder which may be used in concentrated form. They recommend the method of preparing the powder with cream, or, if need be, with water, and particularly emphasise their chief claim in its favour, which is that "Secway" is a living food, which contains all the original enzymes in an unaltered condition.

### MODERN ANTI-ACCIDENT COT.

The committee of the Glasgow Children's Hospital, which the King is to open in July, have decided on the "anti-accident" cots manufactured by Messrs. J. Nesbit-Evans, of Floodgate Street, Birmingham. The cots have no sharp hooks on the pillars, and can be wheeled or fixed in position. The side rails are also designed so as to prevent any possibility of a child's head being caught between the bars. These cots, of which 300 have been ordered, are also in use at the Western Infirmary, Glasgow, the Royal Infirmary, Sheffield, and the General Infirmary, Leeds.

